# Smoking and Idiopathic Pulmonary Fibrosis

**DOI:** 10.1155/2012/808260

**Published:** 2012-02-19

**Authors:** Chad K. Oh, Lynne A. Murray, Nestor A. Molfino

**Affiliations:** ^1^MedImmune, LLC., One MedImmune Way, Gaithersburg, MD 20878, USA; ^2^MedImmune, Ltd., Milstein Building, Granta Park Cambridge, CB21 6GH, UK

## Abstract

Idiopathic pulmonary fibrosis (IPF) is a disease of unknown etiology with considerable morbidity and mortality. Cigarette smoking is one of the most recognized risk factors for development of IPF. Furthermore, recent work suggests that smoking may have a detrimental effect on survival of patients with IPF. The mechanism by which smoking may contribute to the pathogenesis of IPF is largely unknown. However, accumulating evidence suggests that increased oxidative stress might promote disease progression in IPF patients who are current and former smokers. In this review, potential mechanisms by which cigarette smoking affects IPF, the effects of cigarette smoking on accelerated loss of lung function in patients with IPF, key genetic studies evaluating the potential candidate genes and gene-environment (smoking) interaction, diagnosis, and treatment with emphasis on recently closed and ongoing clinical trials are presented.

## 1. Introduction

Idiopathic pulmonary fibrosis (IPF) is defined as a specific form of chronic, progressive fibrosing interstitial pneumonia of unknown cause, occurring primarily in older adults, limited to the lungs, and associated with the histopathologic and/or radiologic pattern of usual interstitial pneumonia (UIP) [1]. Current estimates of IPF prevalence and annual incidence in the United States range from 14 to 42.7 per 100 000 and 6.8–16.3 per 100 000, respectively [2]. There is a high unmet need for novel therapies as conventional therapy has limited efficacy or an unfavorable safety profile and there are no US Food and Drug Administration- (FDA-) approved therapies for IPF treatment. In a recent meta-analysis of observational studies examining environmental and occupational risk factors for IPF, significantly increased risk for IPF was associated with cigarette smoking and exposure [3]. Several environmental exposures also have been associated with increased risk for IPF. Occupational factors, primarily metal and wood dust exposure, adjusted for age and smoking, have been found to be significantly associated with IPF [4, 5]. Among these risk factors, cigarette smoking seems to be the most strongly associated risk factor in both sporadic IPF and familial pulmonary fibrosis [3, 6].

The prevalence of tobacco use in IPF ranges from 41% to 83%, depending on the case definition used in the studies [7, 8]. Current or former smokers have consistently been overrepresented in IPF [9–13]. In one case-control study, smoking was identified as a potential risk factor for the development of IPF (odds ratio [OR] = 1.6) [14]. The overall OR for smoking as a risk factor for IPF was 1.58. There is an independent strong association between smoking and the development of familial interstitial pneumonia of various subtypes including UIP (OR, 3.6; 95% confidence interval, 1.3–9.8) [6]. Recent work suggests that smoking may have a detrimental effect on survival of patients with IPF. Antoniou and colleagues report that life-long nonsmokers have a better outcome than former smokers and the combined group of current and former smokers in IPF in a study of 249 patients, 186 had a smoking history, although pathogenetic differences have yet to be elucidated [15]. Interestingly, in a large study of IPF, King Jr et al. demonstrated that current smokers have a much longer survival than former smokers, an effect that remained statistically significant after adjustment for functional impairment, age, and chest radiographic severity of disease [16]. It is, however, unclear whether ongoing smoking is genuinely protective against disease progression as a “healthy smoker effect” (i.e., smoking cessation prompted by more rapidly progressive disease) cannot be excluded [17, 18].

The mechanism by which smoking may contribute to the pathogenesis of IPF is unknown despite studies suggesting a strong association between the development of IPF and cigarette smoking. Because IPF is recognized as a disease of aging, it has been speculated that smoking may contribute to the development of interstitial lung disease in an age-dependent manner [19]. Also, there is a suggestion that increased oxidative stress in current and former smokers might promote disease progression [20]. Examination of high-resolution computed-tomography (HRCT) scan images has revealed that some patients with IPF, primarily male smokers, may display the combination of emphysematous lesions and pulmonary fibrosis, which has been called combined emphysema and pulmonary fibrosis [21, 22]. These patients develop severe pulmonary hypertension and present decreased survival rate compared with patients with IPF alone. Smoking is a definite risk factor for developing chronic obstructive pulmonary disease (COPD). Some of the underlying fibrotic remodeling changes that occur in lungs of COPD patients exhibit IPF-like pathologies, with remodeling being evident in the interstitial regions [23], but no underlying interstitial lung disease [24, 25]. It is unknown whether this increase is related to the abnormal pathways upregulated in the disease or, as seems to be the case in IPF combined with emphysema, this relationship is not causal but directly associated to smoking [26]. A search for the potential mechanisms by which cigarette smoking affects patients with IPF may provide insights on the development of novel therapeutic targeted agents against smoking-induced progression of IPF.

## 2. Pathogenesis of IPF

At the cellular level, IPF is characterized by fibroblast proliferation and activation, resulting in pronounced extracellular matrix (ECM) deposition [27]. Histologically, fibrotic areas of the IPF lung are adjacent to areas of mononuclear cell infiltration and areas of normal parenchyma. There is considerable epithelial cell apoptosis as well as the abnormal presence of myofibroblasts in the lung parenchyma [28]. Collectively, these cell phenotypic alterations contribute towards the reduced efficiency of gas exchange due to excess ECM deposition, whilst enhancing the contractility of the lung tissue and thus reduced lung tissue compliance.

Fibroblasts are stromal cells that support tissue homeostasis through the generation of ECM during normal wound healing, as well as differentiating into alpha-smooth muscle actin-positive myofibroblasts. In addition to being highly synthetic, myofibroblasts are proposed to contract wound edges together, thus sealing off injured tissue and preventing damage or subsequent infection [29]. During normal wound healing, myofibroblasts apoptose and scar tissue resolves, in part via enzymatic matrix metalloproteinase (MMP) generation [30]. However, in the case of IPF, myofibroblasts persist in the lung, generating even more ECM and further contributing to the underlying scar tissue [31]. These cells also have a heightened fibrotic baseline, producing greater levels of ECM and profibrotic mediators, as well as expressing greater levels of profibrotic receptors such as transforming growth factor- (TGF-) *α* receptors and interleukin- (IL-) 13 receptors [32].

Idiopathic pulmonary fibrosis fibroblasts have a lower threshold for activation and thus can have an augmented profibrotic response to mediators found in the IPF lung [32]. Fibroblasts are activated by a number of processes ranging from physical stretch, to alterations in underlying ECM and directly by soluble mediators including growth factors and reactive oxygen and nitrogen species (RONS) [33]. However, fibroblasts can also be activated by apoptotic debris found at sites of injury and inflammation to generate excess ECM and profibrotic mediators and undergo apoptosis [34].

As well as being highly activated, there is an increase in fibroblast number in the lungs of IPF patients, which in part may be due to a relative resistance to apoptosis [35]. However, the sources of fibroblasts during IPF appear to be altered when compared to healthy nonfibrotic control patients. For example, one process by which fibroblasts have recently been described to arise from is the epithelium. Epithelial abnormalities have been described histologically in IPF, with areas of apoptosis and denuded epithelium prevalent in the fibrotic lung [36].

Epithelial-to-mesenchymal transition (EMT) is a process that occurs normally during early fetal development where a seamless plasticity exists between epithelial and mesenchymal cells [37]. During EMT, epithelial markers including tight junction proteins are downregulated and a concomitant upregulation of mesenchymal markers is observed, with the cells becoming more motile [38, 39]. This phenomenon is well accepted in cancer as a key mechanism that supports tumor progression and metastases [40], and in contrast to developmental EMT, tumorigenic EMT is poorly regulated [41]. In chronic lung diseases, the dual expression of epithelial and mesenchymal markers in the same cells has led investigators to postulate EMT as a mechanism resulting in more ECM-generating cells in the lung [42, 43]. The differentiation of airway epithelial cells has been previously described, for example, type I pneumocytes transitioning into goblet cells, which may in part explain the increase in mucus production in asthma [44–47]. However, the switching of an epithelial cell into a phenotype that moves beyond the original cell's embryonic lineage, that is, into mesenchymal cells, has only recently been hypothesized as a driving factor in fibrosis [39, 48, 49].

Epithelial cells exposed to the prototypic profibrotic growth factor TGF-*α* 1, alone or in combination with other growth factors such as epidermal growth factor, begin the process of EMT by increasing the expression of MMPs [40, 50]. These enzymes will promote basement membrane degradation and cell detachment, allowing for enhanced cell motility. The cells also undergo cytoskeletal changes, as well as altered expression of surface molecules, which allow for the migration and transition of these cells to a mesenchymal phenotype [50, 51]. The idea of EMT promoting the fibrosis observed in IPF is rapidly beginning to evolve [47]. Several recent studies have also shown that EMT occurs in lung epithelial cells both *in vitro* and *in vivo*, supporting the concept of EMT contributing to the fibrosis observed in IPF [47, 52, 53].

Another potential source of lung fibroblasts is the bone marrow. Fibroblasts, traditionally thought to arise from proliferation of resident stromal cells, have been shown to derive from CD45+ collagen-I-producing cells, commonly referred to as “fibrocytes” [54–56]. During *in vitro* culture, circulating CD45+ collagen I+ cells downregulate CD45 and become more adherent [57]. Moreover, in experimental models of lung fibrosis, tracing circulation-derived collagen-I-producing cells confirmed this downregulation of the hematopoietic marker [54]. Fibrocytes have been observed at sites of active fibrosis and increased circulating numbers correlate with mortality in IPF [58]. They are induced by profibrotic mediators such as TGF*β*1 and Th2 cytokines [59]; and express a variety of markers including leukocyte markers (CD45, CD34), mesenchymal markers (collagen I, fibronectin), and chemokine receptors (CCR3, CCR5, CCR7, and CXCR4) [60]. Human and mouse studies have demonstrated that fibrocytes from peripheral blood migrate to skin wound chambers [60, 61]; and bronchial mucosa after antigen challenge [62]. Furthermore, these cells have been reported in disease states with fibrotic pathologies including hypertrophic scars, asthma, and IPF [62–65]. Fibrocytes are pleiotropic and may contribute to fibrogenesis by directly producing collagen, as well as inflammatory cytokines, hematopoietic growth factors, and chemokines [32, 54, 57, 64, 65]. While it was originally thought that fibrocytes promote fibrosis through production of ECM components, it is becoming increasingly hypothesized that their primary role in tissue remodeling may be through secretion of soluble factors [56].

The acute and initial recognition of these foreign mediators initiates a host defense response, resulting in clearance of the noxious agents being mediated by the innate immune system in the lung [66]. One of the first immune cell types in the lung to encounter foreign pathogens is the alveolar macrophage. Macrophages, as well as fibrocytes, derive from monocytes. Following classical activation, as occurs during pathogen recognition and ultimate phagocytosis and clearance, the resultant monocyte-derived cell is the M1 macrophage or “classically activated macrophage [66].” In the fibrotic lung, the predominant macrophage phenotype is the “alternatively activated macrophage,” or the M2 macrophage [67, 68]. Alveolar macrophages are known to remove apoptotic and necrotic debris. In healthy tissue, these cells phagocytose debris and pathogens in a nonphlogistic mechanism, in that downstream inflammation is limited and the inflammatory process is attenuated [69]. In contrast, M2 macrophages are defective at intracellular killing following phagocytosis and dampen the inflammatory response [66]. These macrophages are capable of synthesizing profibrotic mediators, which supports their role in wound healing, but this cell type is inefficient at supporting host defense [66]. The presence of M2-skewed macrophages may explain why patients with IPF are susceptible to exacerbation of disease.

M2 macrophages express elevated levels of scavenger receptors such as macrophage scavenger receptor and mannose receptor C (MRC/CD206) [66, 70]. Assessing circulating primary cells from IPF patients, we have previously described an elevation of CD163+ and CD14+ cells, and M2-associated soluble mediators in the circulation, which was more pronounced in progressive IPF patients. This was suggestive of an overall elevated M2 background in these patients [71]. We have also shown that peripheral blood monocytes from patients with scleroderma-related lung disease display a profibrotic phenotype, characterized by increased CD163 expression and CCL18 expression [72].

Studies of bleomycin-induced fibrosis have assessed either M2 macrophage or fibrocyte number [71, 73]. It is increasingly recognized that there is some overlap between these cell subsets in terms of both function and markers [73]. However, although both fibrocytes and M2 macrophages can be derived from monocytes, they are not completely redundant in function. Recently, using a lung-specific TGF*β*1 overexpression model of lung fibrosis, we have determined that depletion of lung monocyte/macrophages using liposomal clodronate reduced collagen accumulation, but this had no effect on the TGF*β*-induced fibrocyte recruitment [74].

## 3. Mechanisms by which Smoking Contributes to the Pathogenesis of IPF

Cigarette smoke contains particulate matter as well as numerous chemicals including highly toxic RONS [75, 76]. These have been hypothesized to exacerbate lung pathology in a number of ways, including altering both the oxidant/antioxidant balance and the protease/antiprotease balance in the lung; as well as promoting cellular apoptosis and necrosis and generating DNA and lipid intermediates, thus exacerbating inflammation [77–86]. Animal models of cigarette-smoke-induced lung injury are often used for COPD research [84, 87]. Mice challenged with cigarette smoke via passive inhalation exhibit pulmonary neutrophilia after acute (less than 5 days) or chronic (multiple weeks) exposure schedules [87]. The pathology observed in the acute models resolve spontaneously and these models are used to study the inflammation associated with COPD. In contrast, the chronic models generally demonstrate markers of lung destruction, alveolar collapse, and aberrant protease imbalance. There is also increased collagen deposition in chronic smoke models, however, unlike the gold-standard IPF model, the intratracheal bleomycin, the extent of parenchymal remodelling, fibroblastic foci, and fibroproliferation is limited. Interestingly, cigarette smoke exposure has been demonstrated to increase susceptibility of bleomycin-induced lung fibrosis [88].

*In vitro* studies assessing the effects of cigarette smoke have demonstrated that fibroblasts can produce an emphysematous response and increase MMP production [89–92]. The epithelium, which is one of the first cells exposed to the noxious chemicals and particles during smoking, responds in a multitude of ways to these stimuli *in vitro* [93]. These can range from enhanced proinflammatory mediator production, to undergoing apoptosis and necrosis, dependent on the degree of stimulation. The effect of cigarette smoke on EMT has not been determined. Moreover, correlating the extent of epithelial damage in multiple regions of the lungs of any individual patient with smoking history may elucidate any direct relationship.

Cigarette smoke also alters the way in which macrophages recognize foreign particles and pathogens [94]. The debris that is generated from apoptotic and necrotic cells has recently been shown to enhance inflammation and downstream remodeling, through various receptors including toll-like receptors and other pattern recognition receptors including formyl peptide receptors and C-type lectins, which are commonly expressed on alveolar macrophages [95–98]. It still remains to be confirmed whether it is the direct recognition and phagocytosis of particulate matter that results in apoptosis, or the cigarette-smoke-derived RONS that enhances cellular activation and death. Cigarette smoke induces an M2-type macrophage phenotype and this was paralleled during an *in vivo* cigarette smoke model in mice [99]. Therefore, understanding whether cigarette smoke has any direct effect on promoting M2 macrophage expansion may help identify novel pathogenic pathways for smoking at promoting IPF.

Due to the plethora of mediators capable of activating cells, the combination of mediators may have a synergistic effect in the lungs of patients with IPF. Recent evidence suggests that individuals respond differently to cigarette smoke compounds in part through an altered unfolded protein response, resulting in either susceptibility or resistance to the same stimuli [100]. As has been discussed, cigarette smoke contains numerous stimuli and can thus have a multitude of downstream pathogenic responses on the variety of cells found in the IPF lung. Therefore, understanding the underlying environment and fibrotic milieu the cigarette smoke encounters may be central to the extent of the resultant fibrosis and may explain why not all smokers develop IPF or any of the more classically smoking-associated lung diseases including COPD and cancer.

## 4. Genetic Factors

Although the etiology is unknown, a significant percentage of patients with IPF have a familial form of the disease, suggesting genetics might play a role [6, 101]. Based on a genome-wide scan in Finnish families, Hodgson et al. reported a candidate gene for susceptibility for IPF named ELMOD2 (engulfment and motility domain containing 2), a gene of unknown biological function located on chromosome 4q31 [102, 103]. ELMOD2 belongs to the group of six human proteins containing the ELMO domain, which is essential for cellular processes, such as phagocytosis of apoptotic cells and cell migration [104–106]. Pulkkinen et al. later discovered that the major downstream pathways of ELMOD2 are involved in induction of type I and type III interferon mRNA expression, suggesting that ELMOD2 has a role in antiviral responses [107].

Genetic variants within the human telomerase reverse transcriptase (hTERT) or human telomerase RNA components of the telomerase gene are associated with familial pulmonary fibrosis and are present in some sporadic IPF cases. Heterozygous loss-of-function mutations in TERT have been found in up to 15% of kindreds with familial pulmonary fibrosis [108, 109] and in 1% to 3% of sporadic cases [110, 111]. Recently, Diaz de Leon et al. examined the telomere lengths and pulmonary fibrosis phenotype seen in multiple kindreds with heterozygous TERT mutations and reported that TERT mutation carriers have reduced life expectancy [112]. Furthermore, the TERT mutation carriers have higher fractional tissue volume, which is inversely correlated with forced vital capacity and diffusing capacity of the lung for carbon monoxide, and are especially apparent in carriers that smoked, suggesting that smoking is an additional risk factor for loss of lung function in patients with IPF.

A number of candidate genes have been evaluated in search for genetic factors in sporadic cases of IPF. Among others, the following polymorphisms of genes are reported to be associated with increased frequencies in patients with sporadic IPF: genes encoding for cytokines, such as IL-1 a, tumor necrosis factor-*α*, lymphotoxin *α*, IL-4, IL-6, IL-8, IL-10, IL-12, and TGF-ß1 [113–123], *α* 1 antitrypsin and angiotensin-converting enzymes [116, 124], profibrotic molecules (TGF-*β*1), coagulation pathway genes (plasminogen activator inhibitors-1 and -2 and surfactant proteins-A and -B) [125], and MMP-1 [126] polymorphisms, have been reported to have increased frequencies in patients with sporadic IPF. Unfortunately, however, these findings have not been reproduced in subsequent studies.

R131H (rs1801274) polymorphism of the immunoglobulin G receptor Fc*γ*RIIa determines receptor affinity for immunoglobulin G subclasses and is associated with several chronic inflammatory diseases. In a case-control study, Bournazos et al. compared the distribution of Fc*γ*RIIa R131H genotypes in 142 patients with IPF and in 218 controls using allele-specific polymerase chain reaction amplification. These findings support an association between the Fc*γ*RIIa R131H polymorphism and IPF severity and progression, suggesting the involvement of immunological mechanisms in IPF pathogenesis [127].

Xaubet et al. demonstrated that the carriage of double homozygote (GG/CC) at the single nucleotide polymorphism loci of COX2.3050 and COX2.8473 polymorphisms did not increase disease progression but may increase the susceptibility to IPF by approximately 1.4 fold at age 30 and by a smaller fold greater than 1 up to age 66 years [128]. These findings may help to improve understanding of IPF pathogenesis and may lead to the development of new therapeutic strategies.

In the case-control study described above, Bournazos et al. also compared the distribution of Fc*γ*RIIIb NA1/2 polymorphisms using allele-specific polymerase chain reaction amplification and demonstrated that Fc*γ*RIIIb NA1/2 polymorphisms are associated with IPF disease susceptibility. These results support a role for immunological mechanisms contributing to IPF pathogenesis [129].

Currently, the ATS/ERS/JRS/ALAT (American Thoracic Society, European Respiratory Society, Japanese Respiratory Society, and Latin American Thoracic Association) committee does not recommend genetic testing in patients with either familial or sporadic IPF, as part of clinical evaluation because more functional studies that confirm their significance and studies investigating other mutations, associations, and gene-environment relationships are needed [1].

Cigarette smoking has been associated with IPF in case-control studies with patients of different ethnic backgrounds and with patients with familial pulmonary fibrosis, indicating that smoking may confer increased risk for IPF [3, 6]. Checa et al. [126] demonstrated that a putative gene-environment interaction exists between the single nucleotide genetic variant (G/T) at −755 of the MMP-1 promoter and the presence of IPF in smokers. −755 of the MMP-1 promoter creates a potential binding site for transcription factors of the activation protein-1 (AP-1) family and thus may influence the transcriptional responsiveness of the MMP-1 promoter. They found that the TT genotype is increased in cigarette-smoking IPF patients, suggesting that the TT genotype may modulate an interaction with smoking, resulting in an increased susceptibility to develop the disease—a potential gene—environment interaction between the polymorphism and smoking in IPF. Barnes et al. demonstrated that cigarette smoke induces acetylation of histone H4 and decreases histone deacetylase- (HDAC-) 2 activity and expression. Coward et al. [130, 131] also reported that decreased histone acetylation and methylation is responsible for the repression of the antifibrotic cyclooxygenase-2 gene and the potent angiostatic chemokine gamma interferon- (IFN-*γ*-) inducible protein of 10 kDa (IP-10) in lung fibroblasts from patients with IPF. These findings suggest that epigenetic dysregulation involving histone deacetylation and hypermethylation by cigarette smoking may promote fibrotic changes in the lung disease of IPF patients.

## 5. Diagnosis

Diagnosis of an IPF can be achieved with certainty only after the pulmonologist, radiologist, and pathologist have reviewed all of the clinical, radiologic, and pathologic data. The diagnostic criteria for IPF are presented in the ATS/ERS Statement [1]. In the appropriate clinical setting, the presence of a UIP pattern on HRCT is sufficient for the diagnosis of IPF. Thus, the major and minor criteria for the clinical (i.e., nonpathologic) diagnosis of IPF have been eliminated in this new ATS/ERS Statement [1].

Idiopathic pulmonary fibrosis is an idiopathic interstitial pneumonia characterized by UIP histopathology. A confident histopathologic diagnosis of UIP either through a surgical lung biopsy or a characteristic HRCT scan of the chest must be correlated with the clinical findings. The histopathologic criterion is a heterogeneous appearance of areas of fibrosis with scarring and honeycomb change and areas of less affected or normal parenchyma [1]. These changes predominantly affect the subpleural and paraseptal parenchyma. Inflammation is usually mild and consists of a patchy interstitial infiltrate of lymphocytes and plasma cells. The fibrotic zones are composed of dense collagen, proliferating fibroblasts and myofibroblasts, as well as areas of honeycomb change. In the absence of histologic features diagnostic of an alternative condition such as hypersensitivity pneumonitis or autoimmune diseases, such biopsies may be consistent with the diagnosis of IPF.

The typical chest radiograph in IPF shows bilateral basal and peripheral reticular opacities. Progressive fibrosis leads to a reduction in lung volumes and honeycombing, which tends to become less frequent towards the upper lung subpleural zones. In IPF, signs of pulmonary fibrosis include traction bronchiectasis and architectural distortion such as fissure displacement, which can be detected by HRCT [132, 133]. High-resolution computed tomography has been shown to be a highly accurate tool for diagnosis of UIP, with a positive predictive value of 95% to 100% [134]. Therefore, the diagnosis of IPF can be made on the basis of HRCT and clinical evaluation without the need for surgical biopsy.

However, some cases of UIP are difficult to differentiate from fibrotic nonspecific interstitial pneumonia, which may exhibit honeycombing. The most useful finding when differentiating between nonspecific interstitial pneumonia and UIP at HRCT is the greater extent of honeycombing in cases of UIP [135]. The HRCT differential diagnosis of IPF includes pulmonary fibrosis related to connective tissue disease and asbestosis, hypersensitivity pneumonitis, and drug toxicity.

Increasing research evidence indicates that smoking may also result in interstitial lung abnormalities on HRCT [136, 137]. Smokers who develop IPF often have respiratory bronchiolitis-interstitial lung disease and desquamative interstitial pneumonitis changes at HRCT and histopathologic analysis, and patients with desquamative interstitial pneumonitis may develop an HRCT pattern of fibrotic nonspecific interstitial pneumonitis over time [138]. A combination of smoking-related-interstitial lung disease-related HRCT findings, such as ground-glass opacities, cysts, micronodules, septal thickening, and honeycombing, can be seen in the same patient, confounding radiologic classification into a discrete smoking-related entity.

Clinical features of IPF include gradually progressing dyspnea, chronic cough, and bibasilar inspiratory crackles [6]. Digital clubbing is seen in approximately two thirds of patients. Pulmonary function tests usually demonstrate a restrictive defect with reduced lung volumes and diffusing capacity. Idiopathic pulmonary fibrosis should be considered in all adult patients with unexplained chronic exertional dyspnea and commonly presents with cough, bibasilar inspiratory crackles, and finger clubbing [139]. Active smoking in patients with IPF has been shown to be associated with higher values of residual volume and functional residual capacity, as well as with a greater decrease in diffusing capacity of the lung for carbon monoxide [140]. More recently, Washko et al. [23] demonstrated that interstitial lung abnormalities, which were present on about 1 of every 12 HRCT scans, were associated with reduced total lung capacity and a lesser amount of emphysema in smokers using the COPD Gene Study cohorts [137, 141].

## 6. Treatment and Prognosis

The treatment of IPF remains a great challenge as there is no effective therapeutic option. The clinical course is gradual deterioration with a median survival of 2.5 to 3.5 years [142]. Treatment remains largely supportive; prednisone monotherapy or prednisone combined with azathioprine or cyclophosphamide, methotrexate, colchicine, penicillamine, or cyclosporine have been tried but have failed or are not well tolerated by the patient. Supportive care includes supplemental oxygen, vaccination against influenza and pneumococcus, pulmonary rehabilitation, treatment with regular use of immunosuppressant/antioxidant therapy, referral for lung transplant evaluation (when appropriate), and identification and treatment of comorbidities including gastroesophageal reflux disease, sleep disordered breathing, coronary disease, and pulmonary hypertension [143].

Pirfenidone is an oral antifibrotic, anti-inflammatory, and antioxidant agent that inhibits TGF-*βin vitro* [144]. It was approved for use in the management of IPF in Japan in 2008 [145] based on a Japanese phase 3 study involving 267 patients who had been randomly assigned to receive placebo, high-dose (1,800 mg daily), or low-dose (1,200 mg daily) pirfenidone over 52 weeks. The primary endpoint of change in lung vital capacity showed significant preservation of this parameter between the placebo group and the high-dose group (*P* = .0416). Pirfenidone was also studied in two additional multinational, randomized, double-blind, placebo-controlled phase 3 trials (CAPACITY 1 and CAPACITY 2) conducted in IPF patients. The CAPACITY 1 trial enrolled 344 patients and involved a daily dose of 2,403 mg of pirfenidone versus placebo. The CAPACITY 2 trial enrolled 435 patients who were randomly assigned 2 : 2 : 1, respectively, to pirfenidone 2,403 mg daily, placebo, or pirfenidone 1,197 mg daily over a 72-week treatment period. The primary endpoint of change in forced vital was met in CAPACITY 2 (*P* = .001) but not in CAPACITY 1 [146]. Largely based on these data, the European Commission granted marketing authorization for pirfenidone in 2011. In 2010, however, the FDA declined to approve the use of pirfenidone for the treatment of IPF, requesting additional clinical trials.

Given the high morbidity and mortality associated with IPF and the absence of FDA-approved, effective therapies, a wide array of therapeutic approaches are under active clinical investigation. Some key clinical studies evaluating the potential usefulness of the newer agents (e.g., IFN-gamma-1b, pirfenidone, N-acetylcysteine, warfarin, bosentan, etanercept) are summarized in Table 1 (closed studies) and Table 2 (ongoing studies). These studies were cited from the Thomson Pharma database, as the review of all recently completed or ongoing clinical trials exceeds the scope of this review.

Recently, the ATS Guidelines Committee made recommendations of varying strength against the use of most therapies based on data from the completed studies. Treatment recommendations for specific therapies are based on the Grades of Recommendation, Assessment, Development, and Evaluation (GRADE) approach [147]. For example, the recommendation against the use of corticosteroid monotherapy, colchicine, cyclosporine A, combined corticosteroid and immune-modulator therapy, IFN gamma-1b, bosentan, and etanercept for the treatment of IPF is strong.

In the absence of longitudinal data, management of IPF is largely driven by the severity of pulmonary function impairment. A period of observation after smoking cessation is usually warranted to allow spontaneous regression of disease. Relatively good prognosis after smoking cessation with some additive effects of glucocorticosteroids distinguishes smoking-related interstitial lung disease from IPF [148]. As described earlier, life-long nonsmokers have a better outcome than former smokers and the combined group of current and former smokers in IPF [15]. Interestingly, current smokers were found to have a much longer survival than former smokers in a recent large study of IPF [16]. It is not yet clear whether ongoing smoking is genuinely protective against disease progression. Further studies for the potential mechanisms by which cigarette smoking may affect patients with IPF may provide better insight into the effect of smoking on the prognosis of IPF.

## 7. Conclusions

There is a clear need for novel therapies in the treatment of IPF, as conventional therapy has limited efficacy or unfavorable safety profile and no FDA-approved therapies exist. There are a number of ongoing clinical trials targeting fibrogenic, inflammatory, and oxidative pathways. Furthermore, continued effort on better understanding the mechanisms of disease may provide better treatment options in the future. One of the key risk factors for development of IPF, accelerated loss of lung function, and survival is cigarette smoking. The only therapeutic option for these patients with IPF is observation for a period of time after smoking cessation. A search for the potential mechanisms by which cigarette smoking affects IPF and patients with IPF may provide insights on a novel therapeutic target against smoking-induced progression of IPF. A strategy to better understand the effect of smoking on IPF would be to stratify patients based on smoking status and compare the treatment responses between smokers and nonsmokers in the large clinical trials.

## Figures and Tables

**Table 1:** Closed clinical trials in pulmonary fibrosis.

Title: description	Phase	Primary outcomes measure	Trial duration	ClinicalTrials.gov ID
Minocycline Therapy for Lung Scarring in Pts with IPF—A Pilot Study: evaluates the safety and efficacy of minocycline	3	Safety; efficacy	9/13/2005 to (date n/a)	NCT00203697

Interferon-Alpha Treatment of Chronic Cough in COPD and IPF: evaluates the effect lozenges containing interferon-alpha have on coughing	2	Frequency/severity of cough	6/30/2008 to 3/31/2012	NCT00690885

Efficacy and Safety of Oral Bosentan in Pts with IPF: this double-blind, multicenter trial investigated the possible use of bosentan in pts with IPF	2/3	Change in 6MWD	8/31/2003 to 5/31/2010	NCT00071461

Zileuton for the Treatment of IPF: an open-label trial of zileuton compared to azathioprine/prednisone for pts with IPF. Pts underwent a detailed assessment at baseline, followed by 3 mos of posttreatment monitoring for changes in symptoms and physiology	2	Change in LTB4 levels in bronchoalveolar lavage fluid	1/31/2001 to (date n/a)	NCT00262405

Open-Label, Extension Study in Patients with IPF Who Completed Protocol AC-052-321/BUILD 3: an open-label, extension study	3	Long-term safety and tolerability of bosentan	3/31/2008 to 7/31/2010	NCT00631475

Bosentan Use in ILD: BUILD 3 was a prospective, multicenter, randomized, double-blind, parallel-group, placebo-controlled, event-driven, group sequential, superiority study	3	Time to occurrence of disease worsening or death up to end of study	11/30/2006 to 7/31/2010	NCT00391443

Pirfenidone for the Treatment of Pts with PF/IPF: assessed the long-term safety and efficacy of oral pirfenidone in doses of up to 40 mg/kg/day	2	AEs, clinical laboratory tests, directed physical exams	8/31/2003 to 1/31/2011	NCT00080223

Open-Label Study of the Long Term Safety of Pirfenidone in Pts with IPF: an open-label, multicenter, extension study for pts who completed the CAPACITY trials (PIPF-004 and PIPF-006)	3	Obtain safety data	8/31/2008 to 7/31/2012	NCT00662038

Macitentan Use in an IPF Clinical Study: the AC-055B201/MUSIC study compared one dose of macitentan versus placebo in pts with IPF	2	FVC changes	5/31/2009 to 6/30/2011	NCT00903331

Anticoagulant Effectiveness in IPF: tested the effectiveness of warfarin in pts with IPF	3	Time to death, nonbleeding/nonelective hospitalization, or >10% drop in FVC	10/31/2009 to 12/31/2011	NCT00957242

Roll Over Study from 1199.30 BIBF-1120 in IPF: this open-label, non-randomized uncontrolled design trial offers continuation of BIBF-1120 treatment for pts with IPF who have completed a prior clinical trial (1199.30) with that drug. Primary objective is to establish a long-term tolerability and safety profile. Secondary objectives are effects of long-term treatment on survival, safety, and efficacy	2	FVC decline (time frame: 3 years average)	6/30/2010 to 6/30/2015	NCT01170065

IPF: idiopathic pulmonary fibrosis. n/a: not available. COPD: chronic obstructive pulmonary disease. 6MWD: six-minute walking distance. LTB4: leukotriene B4. ILD: interstitial lung disease. BUILD: bosentan use in interstitial lung disease. PF: pulmonary fibrosis. AE: adverse event. MUSIC: macitentan use in an idiopathic pulmonary fibrosis clinical study. FVC: forced vital capacity.

**Table 2:** Ongoing clinical trials in pulmonary fibrosis.

Title: description	Phase	Primary outcomes measure	Trial duration	ClinicalTrials.gov ID
Bosentan in PH in ILD Treatment Study: aims to determine the ability of bosentan to reduce PH in patients with scarring (fibrosing) lung disease	4	Fall in PVR of 20% over 16 wks	4/30/2008 to 8/31/2010	NCT00637065

Treprostinil Therapy for Pts with ILD and Severe PAH: the hypothesis is that IV or SC treprostinil can improve 6MWD, hemodynamics and QOL in pts with ILD and severe secondary PAH	3	6MWD	1/31/2008 to 2/28/2009	NCT00705133

PAH Secondary to IPF and Treatment with Sildenafil: evaluates whether sildenafil improves morbidity and mortality in the peri-lung transplant setting in both IPF cohorts with either resting or exercise PAH	4	6MWD	2/28/2007 to 2/31/2009	NCT00625079

Pilot Study of a Multi-Drug Regimen for Severe PF in HPS: evaluates if pravastatin, losartan, zileuton, N-acetylcysteine, and erythromycin used together can slow the course of PF in pts with HPS	1/2	Survival	4/30/2007 to (date n/a)	NCT00467831

A Study to Evaluate the Safety and Effectiveness of CNTO-888 Administered IV in Subjects with IPF: evaluates safety and efficacy of CNTO-888	2	Pulmonary function and safety	12/31/2008 to 6/30/2012	NCT00786201

Losartan in Treating Pts with IPF: evaluates the safety and efficacy of losartan	n/a	FVC	3/31/2009 to (date n/a)	NCT00786201

Losartan in Treating PF Caused by Radiation Therapy in Pts with Stage I, II, or III NSCLC: evaluates the efficacy of losartan in this pt population	n/a	Change in carbon monoxide diffusing capacity	5/31/2009 to (date n/a)	NCT00880386

Trial of Iloprost in PH Secondary to PF: evaluates safety and efficacy of iloprost	3	Safety; pulmonary arterial pressure; 6MWT	3/31/2007 to 8/31/2007	NCT00439543

Targeting Vascular Reactivity in IPF: evaluates if combination therapy with N-acetylcysteine, sildenafil, and losartan can improve function and exercise tolerance in pts with IPF	2/3	A 6MWT; QOL score	9/30/2009 to 7/31/2013	NCT00981747

Pomalidomide for Cough in Pts With IPF: evaluates the safety and efficacy of pomalidomide over a 12-week duration in the treatment of chronic cough in pts with IPF	2	Cough-related QOL	7/31/2010 to 7/31/2013	NCT01135199

Trial of IW-001 in Pts with IPF: an open-label, multicenter study designed to explore the biological and clinical effects of IW-001	1	Safety and tolerability	9/30/2010 to 9/30/2011	NCT01199887

A Study to Characterize the Safety, PK and Biological Activity of CC-930 in IPF: evaluates the safety and PK profile of CC-930	2	Safety and PK	1/31/2011 to 1/30/2013	NCT01203943

Safety and PK Study with AB-0024 in Pts with IPF: this dose-escalation study evaluates the safety, tolerability, PKs, and pharmacodynamics of AB-0024	1	Safety and PK	12/31/2010 to/31/2012	NCT01242189

A Study of the Safety, Tolerability, PKs, and Pharmacodynamics of IV PRM-151 in Pts with IPF: this study includes multiple doses of IV PRM-151	1b	Safety and tolerability	1/31/2011 to 8/31/2011	NCT01254409

Safety, Tolerability, and Efficacy Study of IPF: evaluates the safety and tolerability of FG-3019 in pts with IPF and the efficacy of FG-3019 for attenuating fibrosis in these pts	2a	Safety and tolerability	12/31/2010 to 12/31/2012	NCT01262001

Safety and Efficacy of QAX-576 in Pts with Progressive IPF: designed to evaluate the safety, tolerability, PKs and efficacy of QAX-576 in pts with rapidly progressive IPF	2	Safety, tolerability, and effect on lung function	12/31/2010 to (date n/a)	NCT01266135

Combined PEX, Rituximab (a chimeric MAb against the protein CD20) and Steroids in Acute IPF Exacerbations: an open-label trial to compare combined plasma exchange, rituximab, and conventional corticosteroid administration on the outcomes of hospitalized pts	1/2	Feasibility and safety	3/31/2011 to (date n/a)	NCT01266317

Safety and Efficacy of BIBF-1120 at High-Dose in IPF Pts:. a prospective, randomized trial that compares BIBF-1120 with placebo	3	Annual rate of decline in FVC	4/30/2011 to 1/31/2014	NCT01335464

PH: pulmonary hypertension. ILD: interstitial lung disease. PVR: pulmonary vascular resistance. IPF: idiopathic pulmonary fibrosis. PAH: pulmonary arterial hypertension. 6MWD: six-minute walk distance. QOL: quality of life. PF: pulmonary fibrosis. n/a: not available. HPS: Hermansky-Pudlak syndrome. FVC: forced vital capacity. NSCLC: non-small cell lung cancer. 6MWT: 6-minute walk test. PK: pharmacokinetic. PEX: plasma exchange. MAb: monoclonal antibody.
